# Loquat Flowers Exceed Leaves: A Less Explored Phenolic Source with Functional Potential

**DOI:** 10.3390/nu18060924

**Published:** 2026-03-14

**Authors:** Ignacio Moratilla-Rivera, Natalia García-Acosta, Jara Pérez-Jiménez, Raquel Mateos

**Affiliations:** 1 Department of Metabolism and Nutrition, Institute of Food Science, Food Technology and Nutrition (ICTAN), Spanish National Research Council (CSIC), 28040 Madrid, Spain; i.moratilla@ictan.csic.es (I.M.-R.); n.garcia@ictan.csic.es (N.G.-A.); 2 Departamento de Genética, Fisiología y Microbiología, Facultad de Ciencias Biológicas, Universidad Complutense de Madrid (UCM), 28040 Madrid, Spain; 3 Departamento de Bioquímica y Biología Molecular, Facultad de Farmacia, Universidad Complutense de Madrid (UCM), 28040 Madrid, Spain; 4 CIBER de Diabetes y Enfermedades Metabólicas Asociadas (CIBERDEM), Instituto de Salud Carlos III (ISCIII), 28029 Madrid, Spain

**Keywords:** *Eriobotrya japonica*, loquat by-products, phenolic compounds, non-extractable polyphenols, HPLC-ESI-QTOF-MS, antioxidant activity, α-glucosidase inhibition, functional ingredients

## Abstract

**Background/Objectives**: Loquat (*Eriobotrya japonica*) is widely cultivated for its fruit, while its aerial by-products remain largely underexploited despite increasing interest in plant-derived bioactive compounds and sustainable food systems. This study comprehensively investigates and compares the phenolic composition and in vitro bioactivities of loquat leaves and flowers to support their potential valorisation as functional ingredients. **Methods**: Extractable and non-extractable polyphenolic fractions were obtained and quantified, and the extractable fraction was further characterised using HPLC-ESI-QTOF-MS. In vitro bioactivity assessment included antioxidant capacity (FRAP and ABTS), glucose dialysis retardation index, and α-glucosidase inhibition. **Results**: Flowers contained significantly higher levels of both extractable and non-extractable polyphenols than leaves. Qualitative and semi-quantitative phenolic profiling, including multivariate analysis, revealed clear compositional differences between the two organs. Flowers showed a higher relative abundance of phenolic acids, as well as the presence of several compounds absent in leaves, such as kaempferol, naringenin-3-*O*-glucoside, and three glycosilated anthocyanins. Flower-derived fractions exhibited consistently higher antioxidant activity across all phenolic fractions than leaf-derived fractions, in agreement with their greater polyphenol content. Regarding antidiabetic activity, leaf samples showed a modest capacity to delay glucose diffusion, whereas this effect was not observed in flowers. In contrast, flower extracts displayed a strong inhibitory effect against α-glucosidase, exceeding that of the reference inhibitor acarbose, while this activity was not detectable in leaf extracts under the experimental conditions. **Conclusions**: These findings support the revalorisation of loquat by-products, particularly flowers, as sustainable sources of bioactive compounds with potential applications in functional foods and health-related products.

## 1. Introduction

Loquat (*Eriobotrya japonica* (Thunb.) Lindl.) is an evergreen tree of Asian origin, widely cultivated in China and Spain for its nutritious fruits. Notably, the production chain generates waste materials such as leaves, flowers, and seeds, which are rich in phytochemicals and therefore represent promising materials for research into their composition and bioactivity, with the aim of supporting their potential valorisation as functional ingredients for food and health-related applications, especially in metabolic disorders [1,2].

Despite the underutilisation of loquat by-products in Western pharmacological or nutraceutical applications, loquat leaves have been traditionally employed in the Chinese Pharmacopoeia for several applications [3]. Beyond their folk use, preclinical studies have shown that leaf extracts reduce oxidative stress and inflammation induced by hyperglycaemia in mice [4] and improve fasting glucose levels, hepatic triglyceride accumulation, body weight gain, and lipid markers in rats fed a high-fat diet [5,6]. These biological effects have been attributed to the presence of bioactive compounds such as triterpenic acids (ursolic acid, oleanolic acid, maslinic acid, tormentic acid), dietary fibre, and polyphenols (isoquercitrin, naringenin, hesperidin) [7]. Among these constituents, polyphenols have attracted particular interest due to their chemical diversity and well-established biological relevance and have therefore been extensively characterised using advanced analytical techniques, including HPLC-DAD-ESI-QToF [5] and UPLC-HRMS [8]. However, the existing literature has focused almost exclusively on the extractable polyphenol fraction, leaving the non-extractable fraction, which is potentially relevant for biological activity within the gastrointestinal tract and after absorption of microbially derived metabolites [9], largely unexplored.

In contrast to the extensive evidence available for loquat leaves, most studies about loquat flowers have focused on the total phenolic content and antioxidant activity [10,11], or on extraction processes rather than on biological activity [12]. To date, only one study has examined the bioactivity of flavones from loquat flowers, reporting a protective effect against acute alcohol-induced liver injury in mice, potentially associated with antioxidant and free-radical scavenging properties [13]. More recently, terpenic profiling under different thermal treatments using UPLC-MS/MS has confirmed the presence and relative stability of compounds in loquat flowers, indicating their potential as a source of bioactive constituents, in addition to contributing to a more detailed understanding of floral composition [14]. Nevertheless, polyphenol fractions in loquat flowers remain poorly characterised, particularly in terms of structural diversity and biological relevance.

Taken together, it remains unclear whether the bioactivity and antidiabetic potential of loquat flowers differ from those of the leaves. Addressing this gap is essential to determine whether loquat flowers represent a distinct and underexplored source of bioactive compounds relevant to metabolic health. Thus, the present study aims, by integrating comprehensive polyphenol profiling using targeted metabolomics with functional assays, to clarify the contribution of both extractable and non-extractable fractions to bioactivity in loquat flowers and leaves. This approach seeks to provide a more complete assessment of their biological potential and to support the sustainable valorisation of loquat by-products within a food and health framework.

## 2. Materials and Methods

### 2.1. Plant Material

**Figure 1:**
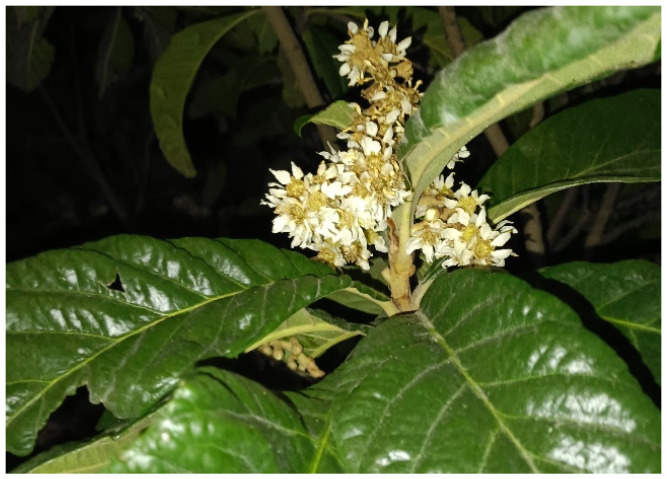
Morphological details of flowers and leaves from *Eriobotrya japonica* specimen.

Plant material was provided by the Agricultural Cooperative of Callosa d’en Sarrià. Leaves and flowers from *Eriobotrya japonica* cultivars (cv. ‘Algerie’) were collected in March 2024 in Alicante (Spain), and morphological characteristics are shown in Figure 1. The samples were collected from three different five-year-old trees grown under identical agronomic conditions (clay soil, drip irrigation at 24 L/day, and fertigation) at coordinates 38°38′58.06″ N and 0°6′15.67″ W. At the time of sampling, the climatic conditions were characterised by an average temperature of 15.8 °C, relative humidity of 54.75%, and a monthly precipitation of 16 m^3^. Flowers and leaves were manually separated prior to processing. Each tissue was subsequently freeze-dried and ground to a particle size of ≤0.5 mm prior to solvent extraction.

### 2.2. Isolation of Phenolic Fractions

#### 2.2.1. Extractable Polyphenols (EPPs)

Extractable polyphenols were obtained following the method described by Pérez-Jiménez et al. (2008) [15]. Briefly, 0.5 g of freeze-dried sample was mixed with 20 mL of methanol:water (50:50, *v*/*v*, pH 2) (PanReac Applichem, Barcelona, Spain) and shaken for 1 h. The mixture was then centrifuged at 3000 rpm for 10 min, and the supernatant was collected. The residue was re-extracted with 20 mL of acetone:water (70:30, *v*/*v*) (PanReac Applichem, Barcelona, Spain) under the same conditions. After a second centrifugation (3000 rpm, 10 min), both supernatants were combined (1:1, *v*/*v*), yielding the extractable polyphenol fraction.

#### 2.2.2. Non-Extractable Polyphenols (NEPPs)

Independent solid residues remaining after EPP extraction were used to obtain two non-extractable polyphenol fractions: hydrolysable polyphenols (HPPs, corresponding to small phenolic acids associated with cell wall by several linkages, including covalent bonds) and non-extractable proanthocyanidins (NEPAs), which are high-molecular-weight proanthocyanidins). The release of both fractions from the food matrix requires harsh chemical conditions, although this may cause the degradation of some structures [16].

HPPs were obtained by adding 20 mL of methanol (PanReac Applichem, Barcelona, Spain) and 2 mL of concentrated H_2_SO_4_ to the residue, followed by incubation at 85 °C under agitation for 20 h. After centrifugation (3000 rpm, 10 min), the supernatant was collected. The remaining residue was washed with distilled water and centrifuged again under the same conditions. All supernatants were combined and adjusted to a final volume of 50 mL, corresponding to the HPP extract [17].

NEPAs were obtained by adapting the method described by Porter et al. (1985) and Zurita et al. (2012) [18,19]. The residue was treated with 10 mL of butanol:HCl (97.5:2.5, *v*/*v*) (PanReac Applichem, Barcelona, Spain) containing 0.7 g/L FeCl_3_ and incubated at 100 °C for 1 h under agitation. After centrifugation (3000 rpm, 10 min), the supernatant was collected. Two additional washings were performed using the same solvent mixture, and all supernatants were combined and adjusted to 50 mL, yielding the NEPA fraction.

### 2.3. Determination of Phenolic Compounds

#### 2.3.1. Total Content of Extractable and Non-Extractable Polyphenols

For EPP and HPP fractions, total content was determined using the Folin–Ciocalteu method [20]. Briefly, 25 μL of extract was mixed with 25 μL of Folin–Ciocalteu reagent (Sigma-Aldrich, Madrid, Spain). After 5 min, 500 μL of Na_2_CO_3_ (75 g/L) (Sigma-Aldrich Madrid, Spain) and 350 μL of distilled water were added. The mixture was shaken and incubated for 60 min, and absorbance was measured at 750 nm. Results were calculated using a gallic acid (Sigma-Aldrich Madrid, Spain) calibration curve prepared in the corresponding solvents for each fraction and expressed as g gallic acid equivalents (GAEs)/100 g dry weight (dw).

For the NEPA fraction, total content was determined using a colorimetric method based on absorbance measurements at 450 and 550 nm. The sum of both absorbances was interpolated against a calibration curve prepared with high-molecular-weight carob proanthocyanidins and expressed as g proanthocyanidin equivalents/100 g dw.

#### 2.3.2. Qualitative and Quantitative Analysis by HPLC-ESI-QTOF

Phenolic profiling was performed exclusively in the EPP fraction using HPLC-ESI-QTOF (Agilent Technologies, Santa Clara, CA, USA). An aliquot of 1 mL of EPP extract was evaporated to dryness under a nitrogen stream. The residue was reconstituted in 200 μL of water (1% formic acid):acetonitrile (50:50, *v*/*v*) (PanReac Applichem, Barcelona, Spain) and vortexed to ensure complete dissolution. The final solution was filtered through a 0.45 μm cellulose acetate syringe filter and transferred to chromatographic vials.

A volume of 5 μL was injected per sample. Separation was performed on a C18 reversed-phase column equipped with a guard column, using a binary mobile phase consisting of solvent A (water) and solvent B (acetonitrile), both containing 0.1% formic acid. The flow rate was set at 0.4 mL/min. The gradient started at 5% B, increased to 30% at 15 min, to 50% over the next 5 min, and to 90% in a further 5 min, which was maintained for 4 min. The system was then returned to initial conditions (5% B) in 1 min and equilibrated for 7 min before the next injection.

Mass spectrometry conditions were as follows: drying gas (nitrogen, 99.9% purity) at 10 L/min and 325 °C; sheath gas at 6 L/min and 250 °C; nebulizer pressure 25 psi; capillary voltage 3500 V; nozzle voltage 500 V; fragmentor voltage 150 V. Data were acquired over an *m*/*z* range of 100–970 in negative ion mode, except for anthocyanins, which were analysed in positive ion mode.

Data processing was carried out using MassHunter Data Acquisition and MassHunter Qualitative Analysis 13.0 software (Agilent Technologies^®^, Santa Clara, CA, USA). Compound identification was performed using commercial standards from Merck (Rhaway, NJ, USA) (protocatechuic acid, 4-hydroxybenzoic acid, kaempferol, kaempferol-3-*O*-glucoside, quercetin, quercetin-3-*O*-glucoside, rutin, caffeic acid, ferulic acid, *p*-coumaric acid, 5-caffeoylquinic acid, catechin, epicatechin, procyanidin B-type dimer, cyanidin, and malvidin-3-*O*-glucoside) or by comparison of accurate mass and MS/MS fragmentation patterns with public databases (e.g., METLIN, MassBank). The level of compound identification was classified according to the Metabolomics Standards Initiative (MSI) guidelines into four confidence levels, including Level 1, confirmed with an authentic standard; Level 2, putatively annotated based on spectral similarity to databases; Level 3, putatively characterised compound classes; and Level 4, unknown compounds.

### 2.4. Antioxidant Capacity

#### 2.4.1. Ferric Reducing Antioxidant Power (FRAP) Assay

The FRAP assay [21] was applied to EPP and HPP fractions due to interference of the NEPA solvent system with the assay chemistry (i.e., butanol medium with aqueous medium). The FRAP reagent was freshly prepared by mixing 25 mL of acetate buffer (0.3 M, pH 3.6), 2.5 mL of TPTZ (10 mM in 40 mM HCl) (Sigma-Aldrich, Madrid, Spain), and 2.5 mL of FeCl_3_·6H_2_O (20 mM) (PanReac Applichem, Barcelona, Spain). For each measurement, 150 μL of FRAP reagent, 15 μL of distilled water, and 15 μL of extract were mixed and incubated at 37 °C for 30 min. Absorbance was recorded at 595 nm. Results were calculated using a Trolox (Sigma-Aldrich, Madrid, Spain). calibration curve and expressed as μmol Trolox equivalents/g dw.

#### 2.4.2. ABTS Radical Cation Scavenging Assay

The ABTS radical scavenging assay [22] (2,2′-azino-bis(3-ethylbenzothiazoline-6-sulfonic acid) diammonium salt) (Sigma-Aldrich, Madrid, Spain). was performed on the EPP and NEPA fractions, as the strong acidic conditions used for the HPP fraction interfered with the assay. The ABTS•^+^ working solution was prepared by dissolving 38 mg of ABTS in 10 mL of 2.45 mM potassium persulfate (Sigma-Aldrich, Madrid, Spain) and allowing the mixture to react overnight under continuous agitation while protected from light.

For the assay, 270 μL of the ABTS•^+^ solution was mixed with 30 μL of the phenolic fraction or the corresponding solvent mixture (blank). Absorbance was recorded continuously for 6 min at 30 °C. The percentage of radical scavenging activity was calculated relative to the blank, which was considered to represent 0% radical inhibition. The scavenging percentages were then interpolated from a Trolox calibration curve as a reference standard. Results were expressed as μmol Trolox equivalents/g dw.

### 2.5. In Vitro Antidiabetic Assays

#### 2.5.1. Glucose Retardation Index

This technique was applied to intact dried samples after drying and grinding, based on methods described by Aguilar-Ávila et al., 2023 [23]. Briefly, 0.5 g of each sample was weighed and suspended in 25 mL of a 200 mM glucose (Sigma-Aldrich, Madrid, Spain). solution. The mixture was transferred into a dialysis membrane (Medicell International, London, UK) with a molecular weight cut-off of 12,000–14,000 Da. The sealed membrane was placed in a beaker containing 200 mL of distilled water maintained at 37 °C. Aliquots (2 mL) of the external medium were collected at 0, 20, 30, 60, 120, and 180 min. These aliquots represented the amount of glucose diffused through the membrane in the presence of the sample. To correctly interpret the results, the assay was also performed under two additional control conditions: (A) in the absence of the sample, to determine the reference glucose diffusion rate (free glucose control); (B) in the absence of glucose, to assess glucose released from the sample itself.

Glucose concentration in all aliquots was determined using a commercial enzymatic kit (Spinreact, Girona, Spain) based on the glucose oxidase–peroxidase (GOD-POD) colorimetric reaction. For this purpose, 50 μL of the commercial reagent was mixed with 1 mL of each aliquot, and absorbance was measured at 505 nm. The glucose dialysis retardation index (GDRI) was calculated for each time point according to the following equation:

$$GDRI\;\left(\%\right)=100-\left[\frac{\left({Abs}_{sample+glucose}\right)-\left({Abs}_{B}\right)}{{Abs}_{A}}\times 100\right]$$

#### 2.5.2. α-Glucosidase Inhibition Index

This method was applied to the EPP fraction only, as the strong acidic conditions present in the HPP fraction and the butanol medium employed for obtaining the NEPA fractions interfered with the enzyme activity.

The reaction mixture consisted of 10 μL of α-glucosidase (1 U/mL in 0.1 M phosphate-buffered saline, PBS, pH 6.9) (Spinreact, Girona, Spain) and 10 μL of the sample or solvent mixture. Subsequently, 200 μL of *p*-nitrophenyl-β-D-glucopyranoside (*p*NPG) (Spinreact, Girona, Spain) was added. Absorbance was measured at 400 nm at time 0 and after 20 min at 37 °C.

The difference between final and initial absorbance was calculated for each well. In the absence of the sample, this value was defined as 0% inhibition. The assay was then repeated using increasing concentrations of the EPP from each sample. Four concentrations showing a linear dose–response relationship were selected, and the data were linearised assuming first-order enzyme kinetics. The IC_50_ value was calculated as the concentration required to inhibit 50% of α-glucosidase activity.

Acarbose, a clinically used α-glucosidase inhibitor, was used as a positive control and reference standard following the same experimental procedure.

### 2.6. Statistical Analysis

Statistical analyses were performed using SPSS software version 22.0 (SPSS Inc., Chicago, IL, USA). Data distribution was first assessed using the Shapiro–Wilk test to evaluate normality. When the assumption of normality was met, differences among groups were analysed by unpaired Student’s *t*-test—the application of multiple testing correction (FDR) did not change the number of significant compounds.

LC–MS data, based on the peak area for each compound, were processed and analysed using MetaboAnalyst (6.0) for multivariate analysis. Features detected exclusively in a single sample type were removed, retaining only those present in both flower and leaf samples in order to avoid model distortion and overfitting, although, at the same time, this may also have caused the loss of relevant information. This exclusion was applied exclusively for multivariate statistical modelling (PCA and volcano analysis) to ensure mathematical comparability between groups and to prevent zero-inflated variables; however, these features were retained for qualitative phytochemical characterisation and descriptive compositional analysis. The remaining features were normalised using probabilistic quotient normalisation (PQN), log_10_-transformed, and Pareto-scaled prior to statistical analysis. After data preprocessing, principal component analysis (PCA) was performed, and the variables contributing most strongly to model separation were identified based on loading values. Finally, univariate analysis was conducted for each feature and visualised using a volcano plot, highlighting those with the largest fold change and lowest *p*-values.

## 3. Results

### 3.1. Total Phenolic Content of EPP and NEPP

The phenolic content in the extractable (EPP) and non-extractable (NEPP) fractions from *E. japonica* leaves and flowers, determined spectrophotometrically, is presented in Table 1. Overall, all samples exhibited a notably high phenolic concentration, and the total phenolic content was greater in the flowers than in the leaves (9.6 vs. 6.7 g/100 g dw, respectively). Notably, the NEPP fraction surpassed the EPP fraction in both sample types, with NEPA identified as the predominant fraction and showing the highest phenolic content in both plant materials.

### 3.2. HPLC-QTOF-MS/MS Identification of Extractable Polyphenols

The phenolic profile of the EPP fraction from *E. japonica* flower and leaf samples was characterised by HPLC–ESI-QTOF-MS/MS. A total of 59 phenolic compounds were identified based on their relative retention times (RTs), accurate mass measurements, MS/MS fragmentation patterns, and comparison with commercial standards when available; among them, five compounds were present only in flower samples, whereas the remaining compounds were detected in both flower and leaf samples. Table 2 summarises the compounds identified, including their RT, molecular formula, theoretical molecular mass, accurate mass of the molecular ion ([M]^+^ for anthocyanins and [M − H]^−^ for the remaining phenolic compounds, analysed in positive and negative ionisation modes, respectively), and MS^2^ fragment ions.

**Figure 2:**
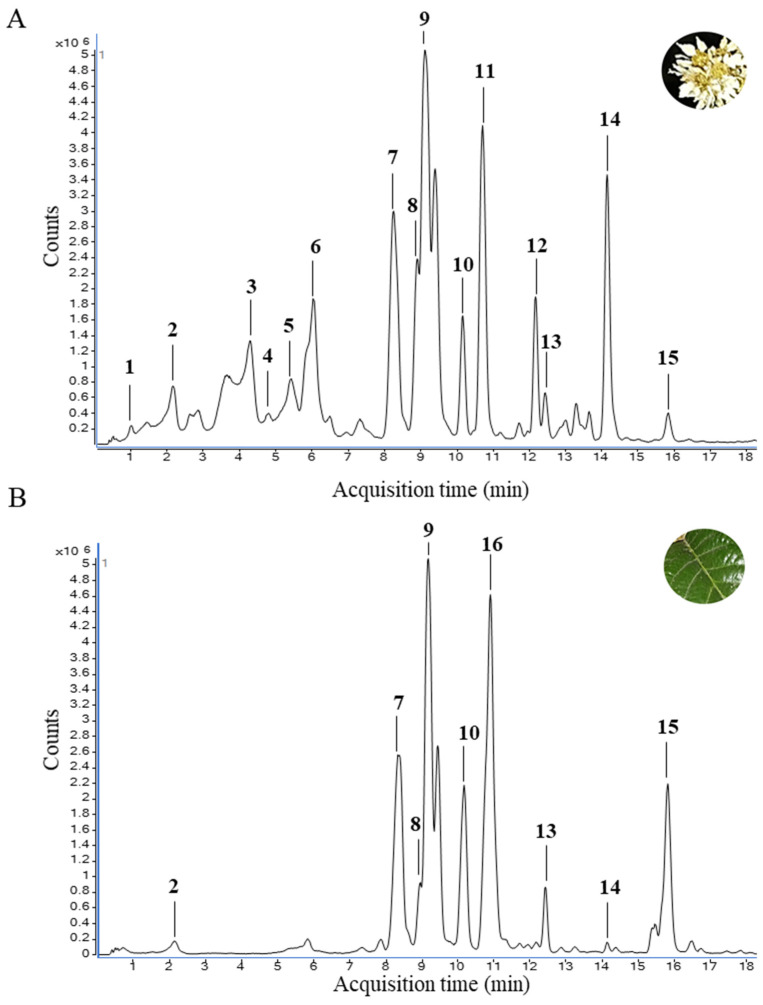
Extracted ion chromatograms (EICs) obtained in negative ion mode (ESI−) for *Eriobotrya japonica* flower (**A**) and leaf (**B**). The EICs were generated using the *m*/*z* values corresponding to the phenolic compounds identified in each sample. For clarity, only selected major signals (representative *m*/*z* values) are depicted in the figure according to their assigned peak number. Salicylic acid (1); Neochlorogenic acid (2); Chlorogenic acid (3); Cryptochlorogenic acid (4); Procyanidin B-type dimer (5); coumaroyl quinic acid (6); Quercetin-3-O-sambubioside (7); Quercetin-3-*O*-rutinoside (Rutin) (8), Isoquercitrin (9); Quercitrin (10); Kaempherol-3-*O*-glucoside (11); Kaempherol-3-*O*-rhamnoside (12); Quercetin-7-O-glucoside (13); Quercetin-4-*O*-glucoside (14); Naringenin-6-C-(2″,4″,6″-*O*-triacetyl)-glucoside (15); Naringenin-6-C-(2″-*O*-acetyl)-glucoside (16).

The extracted ion chromatogram (EIC) revealed the overall phenolic profiles of both matrices, showing a higher number and intensity of signals in the flower samples compared with the leaves. The compounds corresponding to the most intense and representative signals are shown in Figure 2.

Hydroxylated benzoic acids were detected, including monohydroxylated derivatives at positions 1 (salicylic acid), 3, and 4 ([M − H]^−^ at *m*/*z* 137, fragment ion at *m*/*z* 93), as well as the dihydroxylated benzoic acid protocatechuic acid ([M − H]^−^ at *m*/*z* 153, fragment ion at *m*/*z* 109). Regarding hydroxycinnamic acids, three isomers of caffeoylquinic acid [chlorogenic, neochlorogenic, and cryptochlorogenic acids ([M − H]^−^ at *m*/*z* 353, characteristic fragment at *m*/*z* 191)] were identified, together with three isomers of feruloylquinic acid ([M − H]^−^ at *m*/*z* 367, fragment at *m*/*z* 193), two isomers of coumaroylquinic acid ([M − H]^−^ at *m/z* 337, fragments at *m*/*z* 191 and 163), and *o*-coumaric acid ([M − H]^−^ at *m*/*z* 163, fragment at *m*/*z* 119).

Within the flavonol class, glycosylated derivatives of quercetin, kaempferol, and isorhamnetin were predominantly detected (RT 8.4–22.0 min). Notably, kaempferol aglycone was identified exclusively in flower samples, whereas it was absent in leaf samples. Minor subclasses included flavanols, flavanones, and flavones.

Among flavanols, catechin and epicatechin ([M − H]^−^ at *m*/*z* 289, fragment at *m*/*z* 245) as well as procyanidin B-type dimer ([M − H]^−^ at *m*/*z* 577, fragment at *m*/*z* 289) were identified.

The main flavanones corresponded to acetylated naringenin glycosides, in addition to naringenin-3-*O*-glucoside, which was detected only in flower samples ([M − H]^−^ at *m*/*z* 433, fragment at *m*/*z* 271).

Within the flavone group, luteolin ([M − H]^−^ at *m*/*z* 285, fragment at *m*/*z* 133) and its glucoside ([M − H]^−^ at *m*/*z* 447) were detected.

Finally, the anthocyanin profile, derived from the analysis in positive mode, was mainly composed of cyanidin, delphinidin, petunidin, and malvidin glycosides and diglycosides, together with their aglycones. For several anthocyanins, MS/MS spectra could not be acquired due to low signal intensity, and identification was therefore based on accurate mass and retention behaviour (MSI level 2). Despite the lower level of reliance on such identification, it should be mentioned that three anthocyanins were detected exclusively in flower samples, tentatively noted such as cyanidin-3-*O*-(6″-acetyl)-galactoside, petunidin-3,5-diglucoside and malvidin-3,5-diglucoside, eluting at RTs of 5.8, 8.7 and 9.7 min, respectively.

**Figure 3:**
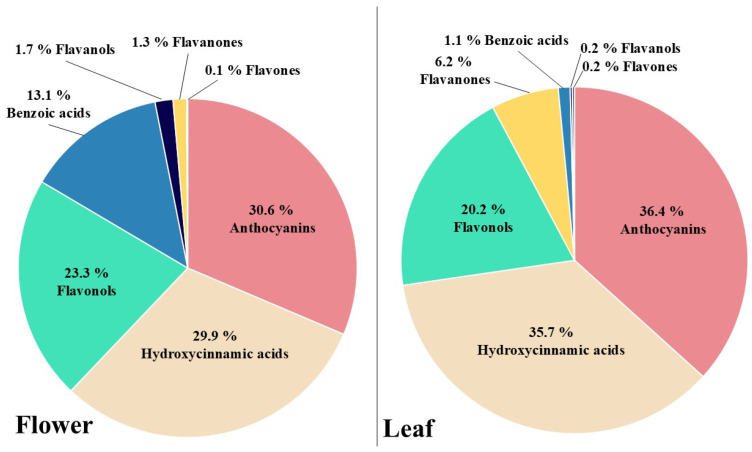
Relative contribution (%) of the main classes of phenolic compounds identified in leaf (**right**) and flower (**left**) samples of *Eriobotrya japonica*, based on HPLC-ESI-QTOF-MS analysis.

Both flowers and leaves contain similar classes of phenolic compounds; however, their relative proportions differed among specific groups, as shown in Figure 3. In both leaves and flowers, anthocyanins, followed by hydroxycinnamic acids and flavonols, were the most abundant classes. However, while in the leaves the three classes corresponded to 92.3% of total phenolic compounds, in the flowers they were 83.8%. The remaining phenolic content corresponded mostly to flavanones in the case of the leaves (6.2%) and to benzoic acids for the flowers (13.1%), while flavanols and flavones were present in both samples at proportions lower than 2%.

### 3.3. Multivariate and Univariate Analysis of Phenolic Compounds

**Figure 4:**
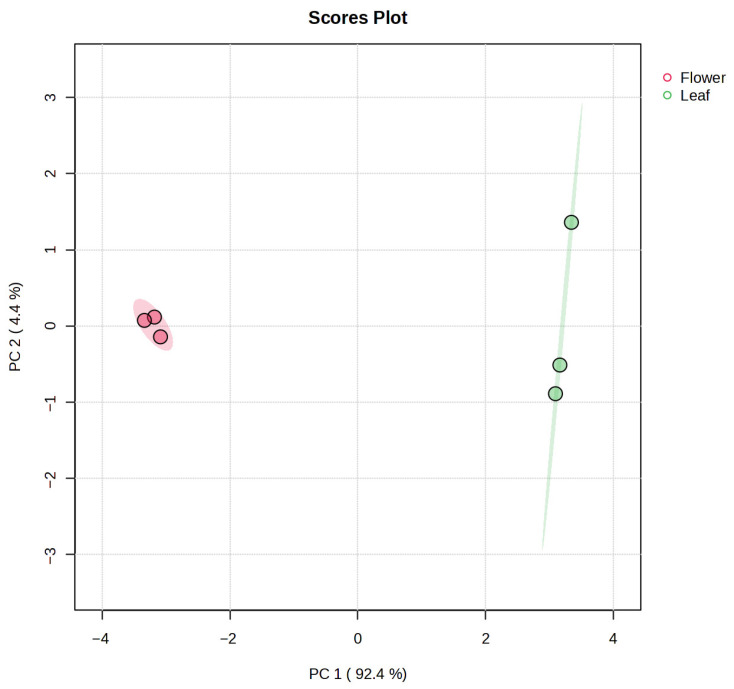
PCA score plot of LC–MS phenolic compounds. Leaf samples are shown in green and flower samples in red.

The LC–MS data were normalised prior to multivariate analysis of the samples, and features detected exclusively in flowers were excluded from the multivariate statistical modelling (PCA and volcano analysis) to prevent overfitting and zero-inflated variables. Importantly, these compounds were retained for qualitative phytochemical characterisation and descriptive analysis, as reported in Section 3.2. This ensured comparability across matrices and avoided zero-inflated features. Normalisation reduced overall inter-sample variability and improved the comparability of metabolite profiles (Supplementary Figure S1). PCA of the metabolite dataset clearly distinguished two groups corresponding to flowers (red) and leaves (green) of *E. japonica* (Figure 4). Notably, the two principal components accounted for more than 97% of the total variance. Principal component 1 (PC1), which accounted for 92.4% of the total variance, clearly separated the two sample types along its axis, indicating marked differences in their chemical profiles. In contrast, PC2 explained only 4.4% of the variance, appearing to contribute minimally to sample discrimination. It should be mentioned that such a strong contribution of PC1, uncommon in this kind of analysis, may be related to the lower number of metabolites included in the model, as compared to the much higher number of features considered in untargeted metabolomics approaches. Another relevant fact was that flower samples exhibited tighter clustering among biological replicates, whereas leaf samples showed greater dispersion between replicates.

**Figure 5:**
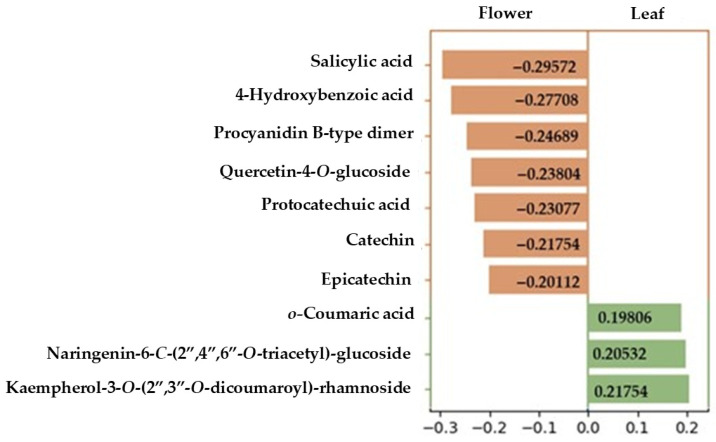
PC1 loading plot showing the ten metabolites with the highest contribution to the discrimination between leaf and flower samples. Compounds with negative loadings (orange) are more strongly associated with flower samples, whereas compounds with positive loadings (green) are more strongly associated with leaf samples. Compounds exclusively identified in flower samples were excluded for the model.

Figure 5 shows the ten compounds with the highest absolute loading values on PC1, representing the strongest contribution to the separation between flower and leaf samples. Flower samples were characterised by a higher contribution as discriminant compounds of phenolic acids (salicylic, protocatechuic, and 4-hydroxybenzoic acids), as well as flavanols (procyanidin B-type dimer, catechin, and epicatechin) and one flavonol, quercetin-4-*O*-glucoside. In contrast, leaf samples were mainly associated with naringenin-6-*C*-(2″,4″,6″-*O*-triacetyl)-glucoside, *o*-coumaric acid, and kaempferol 3-*O*-(2″,3″-*O*-dicoumaroyl) rhamnoside.

**Figure 6:**
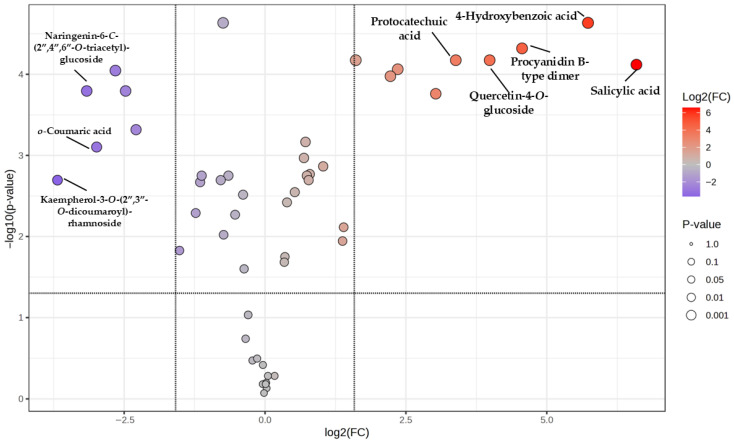
Volcano plot showing differentially accumulated metabolites between leaf and flower samples. The *x*-axis represents log_2_ fold change (log_2_FC) and the *y*-axis represents −log_10_ (*p*-value). Each point corresponds to one metabolite. Colours indicate the magnitude of log_2_ FC, and point size reflects statistical significance. Selected discriminant compounds are labelled. Vertical dashed lines indicate fold change thresholds ([log_2_FC] > 2) and the horizontal dashed line indicates the significance threshold (*p* < 0.05). The log_2_FC was calculated as flower/leaf. Positive log_2_ values indicate metabolites more abundant in flowers, while negative values indicate metabolites more abundant in leaves. Compounds exclusively identified in flower samples were excluded for the model.

The volcano plot (Figure 6) shows the differential accumulation of metabolites between leaves and flowers based on log_2_ fold change (log_2_ FC) and −log_10_ (*p*-value). The log_2_FC was calculated as flower/leaf, so positive log_2_ values indicate metabolites more abundant in flowers, while negative values indicate metabolites more abundant in leaves. Several metabolites exhibited a strong positive log_2_ FC together with high statistical significance, indicating their major contribution to the chemical differentiation between leaf and flower samples. Several metabolites previously identified as major contributors to PC1 separation, including procyanidin B-type dimer, quercetin-4-*O*-glucoside, protocatechuic acid, 4-hydroxybenzoic acid, and salicylic acid, showed strong positive log_2_FC values. Their localisation in the upper right region of the volcano plot (log_2_FC > 3 and −log_10_(*p*-value) > 5) indicates a higher relative abundance in flower samples. Conversely, a subset of metabolites displayed negative log_2_ FC values, suggesting a higher relative abundance in leaf samples. This was the case for kaempferol 3-*O*-(2″,3″-*O*-dicoumaroyl)-rhamnoside, *o*-coumaric acid and naringenin-6-*C*-(2″,4″,6″-*O*-triacetyl)-glucoside.

**Figure 7:**
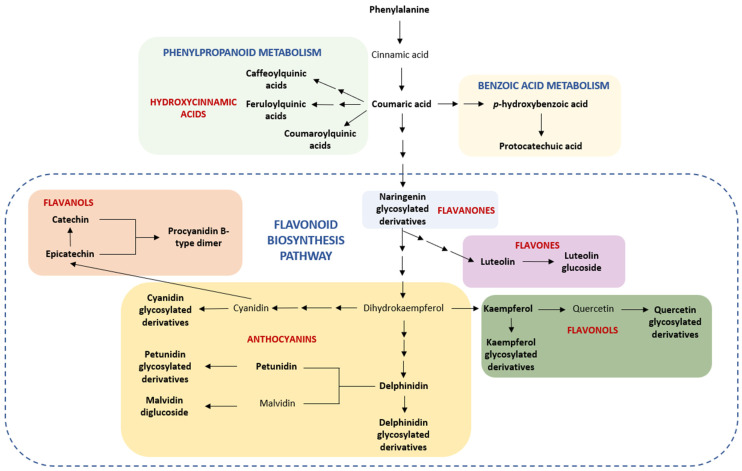
Schematic overview of the main metabolic pathways associated with the phenolic compounds identified in the studied samples. The figure illustrates the metabolic relationships among phenolic compounds without depicting individual enzymatic steps. Single arrows indicate direct conversions, whereas sequential arrows represent multi-step transformations not explicitly detailed in the scheme. The dash box includes the reactions of flavonoid biosynthesis pathway. Compounds detected in the studied matrix are shown in bold.

Finally, Figure 7 presents the key compounds identified in loquat organs mapped onto the general phenylpropanoid pathway, with the aim of illustrating and contextualising their location within the complex metabolic network of vegetal cells.

### 3.4. Antioxidant Capacity

Antioxidant capacity was evaluated in both extractable and non-extractable polyphenol fractions by two complementary methods. Table 3 presents the results obtained using the FRAP assay for the EPP and HPP fractions, and the ABTS assay for the EPP and NEPA fractions (solvent characteristics prevented their application in all the samples). Although the NEPA fraction was the most abundant phenolic fraction in both flower and leaf samples, it exhibited the lowest antioxidant capacity. This apparent discrepancy may be explained by the polymeric nature and reduced redox accessibility of non-extractable proanthocyanidins under chemical assay conditions. In contrast, the EPP and HPP fractions showed a clear association between the phenolic content and antioxidant activity, with the flower EPP fraction displaying the highest antioxidant capacity. Consistent with the total phenolic content results, flower samples exhibited significantly greater antioxidant capacity than leaf samples for all fractions evaluated.

### 3.5. In Vitro Antidiabetic Assays

The antidiabetic effects of the flower and leaf samples were evaluated using two complementary methods: the glucose dialysis retardation index (GDRI), which reflects the ability of the intact plant material to delay glucose diffusion and thus to modulate postprandial glycaemia; and the inhibition of α-glucosidase, the enzyme responsible for the hydrolysis of disaccharides into glucose, which is a therapeutic target of antidiabetic drugs, e.g., miglitol and acarbose.

Flower samples did not exhibit any measurable capacity to retard glucose diffusion through the dialysis membrane at any of the sampling times. On the other hand, leaf samples showed a significant glucose retardation effect at 20 min (14.2% ± 2.0), which progressively decreased at 30 min (9.1% ± 0.9) and 60 min (7.5% ± 2.2), ultimately falling below the detection limit (<5%) at 120 and 180 min (Table 4).

In contrast to the results obtained for the glucose dialysis assay, the EPP extract from flowers exhibited a markedly higher inhibitory capacity against α-glucosidase (IC_50_: 0.48 mg/mL) compared with the corresponding leaf extract, for which inhibition could not be reliably determined under the experimental conditions. Notably, the flower EPP extract showed an IC_50_ value approximately tenfold lower than that of the reference inhibitor acarbose (4.76 mg/mL) (Table 5).

Therefore, leaf and flower samples exhibited, based on in vitro procedures, measurable but distinct potential to modulate postprandial hyperglycaemia through different and complementary mechanisms, namely glucose diffusion retardation by the intact leaf matrix and enzymatic inhibition by extractable polyphenols from flowers.

## 4. Discussion

The present work arose from the interest in re-evaluating loquat (*Eriobotrya japonica*) by-products as sustainable sources of bioactive compounds with potential applications in the food and health sectors [2]. Previous research has focused almost exclusively on the leaves, whereas flowers have been scarcely studied. In this study, we assessed whether the loquat flowers display a phenolic profile, both extractable and non-extractable, and bioactivity comparable, or even superior to, that of the leaf, providing new insight into its potential valorisation.

Our results demonstrate that the non-extractable fraction contains a higher polyphenol content than the EPP fraction in loquat, making a substantial contribution to the total phenolic pool of both leaves and flowers. It should be mentioned that, although the Folin-Ciocalteu method used for the analysis of EPP and HPP total content is subjected to some interferences, which may lead to a certain overestimation of the values, it is the most common assay for a first estimation of the polyphenol content, with a clear relationship with the individual polyphenol content, and is recommended for comparative purposes [15]. In this sense, the values obtained here were in the same range, for instance, as those previously reported for the EPP fraction in loquat flowers (3.6%) [11].

The most relevant fact regarding the total polyphenol content is the high contribution of NEPPs, particularly NEPAs, to the total polyphenol content in loquat flowers and leaves. It should be mentioned that the Porter method, used for determining the total NEPA content, is a specific assay which has been considered as a good estimation of these compounds [15]. Overall, these findings are consistent with existing evidence indicating that plant-derived foods contain substantially greater amounts of non-extractable polyphenols than EPPs [24]. Although bibliographic data regarding NEPPs remain limited, it is noteworthy that flowers and leaves showed substantial levels in NEPA and HPP fractions. In this regard, a study evaluating the content of HPPs in dried fruit peels reported banana peel as the sample with the highest value, reaching levels (~1.9%) comparable to those obtained in the present work [25]. In the same study, most fruit peels exhibited lower NEPA contents, including apple (0.32%), nectarine (0.27%) or pear (0.25%), than those observed here for both loquat tissues. However, despite its quantitative relevance, this fraction is frequently overlooked in most studies, leading to a systematic underestimation of the phenolic content and bioactive potential of plant matrices.

Although this study did not include in vitro antidiabetic bioactivity experiments for the NEPP fraction, the inclusion of this fraction is particularly relevant from a health perspective, especially at the gastrointestinal level. NEPPs largely escape digestion in the upper gastrointestinal tract and reach the colon, where they undergo microbial fermentation, giving rise to bioactive metabolites capable of modulating oxidative, inflammatory, and metabolic processes [26]. Future studies should therefore focus on elucidating the contribution of NEPPs from loquat flowers and leaves to colonic bioactivity and gut microbiota modulation, an area for which evidence remains scarce.

Phytochemical characterisation revealed that loquat leaf and flower extracts exhibited abundant and diverse phenolic profiles. Regarding the polyphenol content, the higher concentrations found in the flowers may be related to the known roles of polyphenols in floral tissues (specific to them), such as their involvement in pollinator attraction and in the protection of the germline against oxidative stress and UV radiation; In addition, the polyphenol content changes throughout the blooming process, being higher in the early bud stage [27].

As regards to the qualitative profile of phenolic compounds, both organs shared a common metabolic core dominated by anthocyanins, flavonols, and hydroxycinnamic acids (Figure 3). However, clear differences were observed in minor compounds, with flowers showing higher levels of phenolic (particularly benzoic acids) and flavanols, whereas leaves were characterised by a greater abundance of flavanones and anthocyanins. Notably, five compounds (kaempferol and the tentatively identified naringenin-3-*O*-glucoside, cyanidin-3-*O*-(6″-acetyl)-galactoside, malvidin-3,5-diglucoside, and petunidin-3,5-diglucoside) were detected exclusively in flower samples. These differences may be explained by the organ-specific regulation of flavonoid biosynthesis and glycosylation pathways [28]. In the case of kaempferol, the presence of its glycosylated derivatives in leaves suggests that the aglycone may act as a transient intermediate undergoing rapid glycosylation. The combined HPLC–QTOF-MS/MS approach together with multivariate analysis enables the integration of complementary information to identify organ-specific compounds, as well as those that, although shared among tissues, display markedly differential abundance patterns.

Interestingly, our profiling expanded the phenolic diversity previously reported in loquat by capturing a broader range of glycosylated and acylated conjugates. This broader coverage may be partly attributed to the use of low-temperature extraction which favours the preservation of labile phenolic structures [29]. Previous studies [5,8] have identified compounds overlapping with our findings, including chlorogenic acid, kaempferol glycosides, rutin, isoquercitrin, procyanidins, and specific structures such as naringenin-6-*C*-(2″,4″,6″-*O*-triacetyl)-glucoside in leaf samples [5,8]. Although quinic acid has been previously reported it was not detected as a free compound in our extracts, but rather esterified as part of more complex molecular structures. This may be partly explained by the high temperature conditions (70 °C) reported in the literature [5] which break the original conformation of hydroxycinnamic acid esters. In contrast, only a limited number of phenolic compounds have been previously characterised in loquat flowers, mainly chlorogenic, ferulic, and caffeic acids from decoction-based preparations (60–90 °C) [30]. Such thermal processing may promote the degradation or structural modification of native phenolic constituents, highlighting the critical importance of mild extraction conditions and high-resolution analytical approaches. Overall, this study provides a comprehensive phytochemical characterisation of loquat tissues, defining organ-specific phenolic fingerprints and revealing an underexplored structural diversity of flavonoids, particularly anthocyanins, through an integrated analysis of multiple phenolic classes and conjugation patterns. Complementary pathway mapping further enabled the integration of these metabolites into putative metabolic pathways, providing a systems-level perspective of their biosynthetic relationships (Figure 7).

Although numerous studies have described the biological activity of flowers and leaves when evaluated separately, this study provides a direct and systematic in vitro comparison between both organs. The antioxidant activity results showed a consistent pattern across all phenolic fractions evaluated, with flowers exhibiting higher antioxidant capacity than leaves (Table 3), in line with their higher TPC. Nevertheless, despite being the most abundant fraction, NEPAs displayed lower antioxidant activity than the EPP fraction, indicating that NEPPs may be less reactive under the conditions of in vitro chemical assays. In this context, previous research on phenolic extracts of loquat leaves found that the butanol fraction (derived from a previous extraction with 80% methanol) presented the highest TPC and the strongest antioxidant activity, suggesting that less polar compounds tend to contribute more strongly to measured bioactivity [31]. This variability likely reflects the influence of factors beyond phenolic composition alone, including the developmental stage, genetic background, and postharvest processing, which may substantially modulate the chemical profile and bioactivity and contribute to discrepancies across studies [14,32].

Antidiabetic activity was assessed in vitro through two complementary intestinal mechanisms: the delay of glucose absorption and inhibition of α-glucosidase. Leaf samples showed a measurable effect on glucose absorption, whereas this effect was not observed in flowers (Table 4). This contrasts with the antioxidant activity results and suggests that the delay in glucose absorption is more closely associated with constituents different from phenolic compounds, mainly dietary fibre. In this sense, a study on the in vitro bioactivities of polysaccharides from leaves found that the key aspects regarding their ability to bind biliary acids or cholesterol were a high molecular weight and high degree of esterification and not the content of associated phenolic compounds [33]. By contrast, the EPP fraction from leaves did not exhibit α-glucosidase inhibitory activity, while flower extracts showed strong inhibition, even exceeding that of acarbose (Table 5). However, such comparisons should be interpreted cautiously, as acarbose is a pure compound, which has passed all required steps to be approved as an effective drug, whereas the extract represents a complex phytochemical mixture evaluated under in vitro conditions, and for which a long evaluation process would still be needed. Thus, the present results for leaves differ from previous studies, which have reported α-glucosidase inhibitory activity in loquat leaf extracts, particularly ethanolic extracts that recover not only phenolic compounds but also triterpenic acids [5], which are well-documented contributors to α-glucosidase inhibition. In the present study, flavonoid composition may further explain the differential α-glucosidase inhibitory activity observed between leaves and flowers. In particular, flavonoids such as quercetin and kaempferol show higher inhibitory activity in their aglycone forms than when glycosylated [30]. Here, kaempferol was the only aglycone flavonoid detected and was exclusively identified in flower samples, potentially explaining their greater inhibitory capacity, although this should be confirmed by molecular docking studies. In this sense, these kinds of studies have suggested that other phenolic compounds present in the samples, such as benzoic acid and flavanols, exert α-glucosidase inhibitory effects [34,35]. This is particularly relevant given that flowers contained higher levels of these two subcategories compared with leaves, which may further contribute to their enhanced inhibitory capacity.

Finally, the in vitro antidiabetic effects observed in this study, together with future research on the possible systemic effects and additional contribution of NEPPs, could contribute to a growing body of evidence suggesting that loquat flowers may play a preventive role in metabolic diseases, pending confirmation in in vivo and clinical studies, similar to what has been documented for loquat leaves in preclinical models [5,6]. Accordingly, loquat flowers emerge as a promising nutraceutical candidate, in line with the growing interest in edible flowers as functional ingredients [36], that merits further evaluation in future in vivo and clinical studies to determine their true potential.

Importantly, future research should address the optimisation of the dosage and mode of administration, particularly to bridge the gap between the polyphenol-rich extract evaluated under controlled in vitro conditions and its potential application in real dietary or nutraceutical contexts. While loquat flowers exhibited superior antioxidant capacity in the present study when compared with leaves, loquat flower processing generates a smaller amount of residual biomass, which may limit large-scale exploitation. Rather than replacing leaf-based applications, this limitation positions flowers as a complementary, value-added source of bioactive compounds, which could be combined with leaves to enhance the overall sustainability and value of the loquat industry.

Overall, this study provided a first integrative approach towards loquat flowers and leaves, considering their phenolic compound content and profile, together with an in vitro assessment of several biological activities. However, several limitations may be mentioned: a lack of complete structural analysis for all polyphenol fractions; and consideration of neither bioavailability and toxicological parameters, particularly assessing realistic doses. These aspects should be explored in further studies which, ultimately, should be based on in vivo models.

## 5. Conclusions

This study demonstrates that loquat aerial by-products, particularly flowers, repre-sent a valuable and underutilised source of bioactive compounds. Based on colorimetric assays, flowers and HPLC-ESI-QTOF-MS analysis showed that leaves and flowers exhibited a higher content of both extractable and non-extractable polyphenols than leaves; displayed a distinct phenolic profile, clearly separated by multivariate analysis; and were characterised by the presence of specific flavonoids and anthocyanins not detected in leaves. These compositional differences seem to have been reflected in their biological activity, as flower extracts showed superior antioxidant capacity and a pronounced α-glucosidase inhibitory effect, whereas leaves exhibited only a modest effect on glucose diffusion. Overall, these findings tend to support the revalorisation of loquat flowers as promising ingredients for the development of functional foods and nutraceuticals, together with leaves, within the context of supporting an integrated strategy for the valorisation of loquat by-products within a sustainable by-product management context. However, these results focus primarily on composition and on rather limited bioactivity. They should therefore be complemented by future research addressing aspects such as the bioavailability of their constituents, their stability and storage conditions, and their effects in preclinical and clinical models, in order to provide stronger evidence of their mechanisms of action.

## Figures and Tables

**Table 1:** Phenolic content of EPP and NEPP (HPP and NEPA) in leaves and flowers of *E. japonica*. Results are expressed as g/100 g dw (mean ± standard deviation). Statistical comparisons between leaves and flowers were performed using an unpaired Student’s *t*-test (* *p* < 0.05). Extractable polyphenols (EPP); dry weight (dw); hydrolysable polyphenols (HPP); non-extractable proanthocyanidins (NEPA).

	EPP (g/100 g dw)	HPP (g/100 g dw)	NEPA (g/100 g dw)	Total (g/100 g dw)
Leaf	1.0 ± 0.1	1.8 ± 0.1	3.9 ± 0.2	6.7 ± 0.2
Flower	2.7 ± 0.1 *	1.8 ± 0.2 *	5.1 ± 0.3 *	9.6 ± 0.4 *

**Table 2:** Identification of phenolic compounds detected in the EPP fraction of *E. japonica* leaves and flowers by HPLC-ESI-QTOF-MS/MS. Compounds were identified based on retention time (RT), accurate mass, quasimolecular ion [M − H]^−^/[M]^+^ ([M]^+^ for anthocyanins and [M − H]^−^ for the other compounds), MS/MS fragmentation patterns, and comparison with commercial standards when available. MSI identification levels are indicated according to Metabolomics Standards Initiative criteria. F, flower; L, leaf. Elution order of the different isomers was assigned based on the existing literature performing RP-HPLC analysis.

Identified Compound	RT (min)	Molecular Formula	Molecular Mass	[M − H]^−^/[M]^+^	MS^2^ Fragments (*m*/*z*)	Sample	MSI Identity Levels
Benzoic acids							
Salicylic acid	0.9	C_7_H_6_O_3_	138.0317	137.0244	93	F; L	2
Protocatechuic acid	1.6	C_7_H_6_O_4_	154.0266	153.0193	109	F; L	1
3-Hydroxybenzoic acid	2.6	C_7_H_6_O_3_	138.0317	137.0244	93	F; L	2
4-Hydroxybenzoic acid	2.8	C_7_H_6_O_3_	138.0317	137.0244	93	F; L	1
Hydroxycinnamic acids							
3-*O*-Caffeoylquinic acid (Neochlorogenic acid)	2.1	C_16_H_18_O_9_	354.0951	353.0878	191; 179	F; L	2
*o*-Coumaric acid	3.4	C_9_H_8_O_3_	164.0473	163.0401	119	F; L	2
Coumaroylquinic acid (isomer 1)	3.5	C_16_H_18_O_8_	338.1002	337.0929	191; 163	F; L	3
5-*O*-Caffeoylquinic acid (Chlorogenic acid)	4.3	C_16_H_18_O_9_	354.0951	353.0878	191; 179	F; L	1
4-*O*-Caffeoylquinic acid (Cryptochlorogenic acid)	4.8	C_16_H_18_O_9_	354.0951	353.0878	191; 173	F; L	2
Coumaroylquinic acid (isomer 2)	6.1	C_16_H_18_O_8_	338.1002	337.0929	191; 163	F; L	3
3-*O*-Feruloylquinic acid	7.1	C_17_H_20_O_9_	368.1107	367.1035	193; 191	F; L	2
5-*O*-Feruloylquinic acid	7.7	C_17_H_20_O_9_	368.1107	367.1035	191; 173	F; L	2
4-*O*-Feruloylquinic acid	8.1	C_17_H_20_O_9_	368.1107	367.1035	193; 173	F; L	2
Flavonols							
Quercetin-3-*O*-sambubioside	8.4	C_26_H_28_O_16_	596.1377	595.1305	300	F; L	2
Quercetin-3-*O*-neohesperidoside	8.7	C_27_H_30_O_16_	610.1534	609.1461	300; 301	F; L	2
Quercetin-3-*O*-rutinoside (Rutin)	9.0	C_27_H_30_O_16_	610.1534	609.1461	300	F; L	2
Quercetin-3-*O*-glucoside (Isoquercitrin)	9.5	C_21_H_20_O_12_	464.0955	463.0882	301; 300	F; L	1
Kaempferol-3-*O*-glucoside-7-*O*-rhamnoside	9.6	C_27_H_30_O_15_	594.1585	593.1512	285; 430; 447	F; L	2
Isorhamnetine-3-*O*-rutinoside	9.7	C_28_H_32_O_16_	624.1690	623.1618	315; 299	F; L	2
Kaempferol-3-*O*-rutinoside	9.8	C_27_H_30_O_15_	594.1585	593.1512	284; 285	F; L	2
Quercetin-3-*O*-xyloside	10.1	C_20_H_18_O_11_	434.0849	433.0776	300; 301	F; L	2
Quercetin-3-*O*-rhamnoside (Quercitrin)	10.2	C_21_H_20_O_11_	448.1006	447.0933	300; 301; 284	F; L	2
Kaempferol-3-*O*-neohesperidoside	10.4	C_27_H_30_O_15_	594.1585	593.1512	285	F; L	2
Isorhamnetine-3-*O*-glucoside-7-*O*-rhamnoside	10.5	C_28_H_32_O_16_	624.1690	623.1618	315; 299	F; L	2
Kaempferol-3-*O*-glucoside	10.7	C_21_H_20_O_11_	448.1006	447.0933	285	F; L	1
Isorhamnetine-3-*O*-galactoside	10.8	C_22_H_22_O_12_	478.1111	477.1038	315; 314	F; L	2
Isorhamnetine-3-*O*-glucoside	11.1	C_22_H_22_O_12_	478.1111	477.1038	314; 271	F; L	2
Kaempferol-3-*O*-rhamnoside	12.2	C_21_H_20_O_10_	432.1056	431.0984	285	F; L	2
Quercetin-7-*O*-glucoside	12.4	C_21_H_20_O_12_	464.0955	463.0882	301; 300	F; L	2
Kaempferol	13.8	C_15_H_10_O_6_	286.0477	285.0405	257	F	1
Quercetin-4-*O*-glucoside	14.1	C_21_H_20_O_12_	464.0955	463.0882	301; 300	F; L	2
Kaempferol 3-*O*-(2″, 3″-dicoumaroyl) rhamnoside	22.0	C_39_H_32_O_14_	724.1792	723.1719	285	F; L	2
Flavanols							
Catechin	3.8	C_15_H_14_O_6_	290.0790	289.0718	245	F; L	1
Procyanidin B-type dimer	5.4	C_30_H_26_O_12_	578.1424	577.1351	289	F; L	2
Epicatechin	5.9	C_15_H_14_O_6_	290.0790	289.0718	245	F; L	1
Flavanones							
Naringenin-6-C-(2″-*O*-acetyl)-glucoside	10.9	C_23_H_24_O_11_	476.1319	475.1246	314; 271	F; L	2
Naringenin-3-*O*-glucoside	12.7	C_21_H_22_O_10_	434.1213	433.1140	271	F	2
Naringenin-6-C-(2″, 4″, 6″-*O*-triacetyl)-glucoside	15.9	C_27_H_28_O_13_	560.1530	559.1457	271	F; L	2
Flavones							
Luteolin-*O*-glucoside	11.7	C_21_H_20_O_11_	448.1006	447.0933	285	F; L	2
Luteolin	16.8	C_15_H_10_O_6_	286.0477	285.0405	133	F; L	2
Anthocyanins							
Cyanidin-3-*O*-(6″-acetyl)-galactoside	5.8	[C_23_H_23_O_12_]^+^	491.1190	491.1184	-	F	2
Delphinidin-diglucoside (isomer 1)	8.0	[C_27_H_31_O_17_]^+^	627.1561	627.1556	-	F; L	3
Cyanidin-3-*O*-(6″-acetyl)-glucoside	8.2	[C_23_H_23_O_12_]^+^	491.1190	491.1184	-	F; L	2
Delphinidin-3-*O*-sambubioside	8.5	[C_26_H_29_O_16_]^+^	597.1456	597.1450	303	F; L	2
Petunidin-3,5-diglucoside	8.7	[C_28_H_33_O_17_]^+^	641.1718	641.1712	-	F	2
Cyanidin-3-*O*-sophoroside	9.1	[C_27_H_31_O_16_]^+^	611.1612	611.1607	287	F; L	2
Cyanidin-3,5-diglucoside	9.2	[C_27_H_31_O_16_]^+^	611.1612	611.1607	287	F; L	2
Delphinidin	9.3	[C_15_H_11_O_7_]^+^	303.0556	303.0499	-	F; L	2
Delphinidin-diglucoside (isomer 2)	9.4	[C_27_H_31_O_17_]_+_	627.1561	627.1556	-	F; L	3
Cyanidin-*O*-glucoside (isomer 1)	9.7	[C_21_H_21_O_11_]^+^	449.1084	449.1074	287	F; L	3
Malvidin-3,5-diglucoside	9.7	[C_29_H_35_O_17_]^+^	655.1874	655.1869	-	F	2
Cyanidin-3-*O*-sambubioside	9.8	[C_26_H_29_O_15_]^+^	581.1506	581.1501	287	F; L	2
Petunidin-rhamnoside-glucoside	10.0	[C_28_H_33_O_16_]^+^	625.1769	625.1763	317	F; L	2
Cyanidin-rhamnoside-glucoside	10.1	[C_27_H_31_O_15_]^+^	595.1663	595.1657	287	F; L	2
Delphinidin-xylose/Delphinidin-arabinose	10.2	[C_20_H_19_O_11_]^+^	435.0927	435.0922	303	F; L	2
Cyanidin-*O*-glucoside (isomer 2)	10.4	[C_21_H_21_O_11_]^+^	449.1084	449.1074	287	F; L	3
Cyanidin-3-*O*-rutinoside	10.6	[C_27_H_31_O_15_]^+^	595.1663	595.1657	287	F; L	2
Petunidin-3-*O*-rutinoside	10.7	[C_28_H_33_O_16_]^+^	625.1769	625.1763	317	F; L	2
Petunidin-*O*-galactoside (isomer 1)	10.9	[C_22_H_23_O_12_]^+^	479.1190	479.1184	317	F; L	3
Petunidin	11.2	[C_16_H_13_O_7_]^+^	317.0661	317.0656	257	F; L	2
Petunidin-*O*-galactoside (isomer 2)	11.2	[C_22_H_23_O_12_]^+^	479.1190	479.1184	317	F; L	3

**Table 3:** Antioxidant capacity of EPP, HPP and NEPA fractions in leaves and flowers of *E. japonica*. The results are expressed in μmol Trolox equivalents/g dw (mean ± standard deviation). Statistical comparisons between leaves and flowers were performed using an unpaired Student’s *t*-test (* *p* < 0.05). Extractable polyphenols (EPP); dry weight (dw); hydrolysable polyphenols (HPP); non-extractable proanthocyanidins (NEPA).

	FRAP EPP (μmol Trolox/g dw)	ABTS EPP (μmol Trolox/g dw)	FRAP HPP (μmol Trolox/g dw)	ABTS NEPA (μmol Trolox/g dw)
Leaf	62.1 ± 5.4	28.7 ± 0.6	104.6 ± 10.2	14.3 ± 1.0
Flower	210.1 ± 13.4 *	139.1 ± 13.2 *	142.0 ± 17.3 *	36.7 ± 1.8 *

**Table 4:** Glucose dialysis retardation index (GDRI) at different times for leaves and flowers of *E. japonica*. The results are expressed as percentage (%) (mean ± standard deviation). Statistical comparisons between leaves and flowers were performed using an unpaired Student’s *t*-test. Not detected (n.d.).

	20 min GDRI (%)	30 min GDRI (%)	60 min GDRI (%)	120 min GDRI (%)	180 min GDRI (%)
Leaf	14.2 ± 2.0	9.1 ± 0.9	7.5 ± 2.2	<5	<5
Flower	n.d.	n.d.	n.d.	n.d.	n.d.

**Table 5:** Inhibitory concentration (IC_50_) of α-glucosidase of extractable polyphenolic fractions (EPP) from loquat leaves and flowers. Acarbose was used as a positive control. The results are expressed as mg/mL (mean ± standard deviation). Statistical comparisons were performed using an unpaired Student’s *t*-test (* *p* < 0.05). Not detected (n.d.).

	IC_50_
Leaf	n.d.
Flower	0.48 ± 0.04 *
Acarbose	4.76 ± 0.08

## Data Availability

Data will be available on request.

## Supplementary Materials

The following supporting information can be downloaded at: https://www.mdpi.com/article/10.3390/nu18060924/s1, Figure S1: Distribution of metabolomic features before and after PQN normalization, log10 transformation, and Pareto scaling, visualized by boxplots and kernel density plots across all samples.
