# Supplementary material for: Loquat Flowers Exceed Leaves: A Less Explored Phenolic Source with Functional Potential
Source: Nutrients. 2026 Mar 14;18(6):924. doi: 10.3390/nu18060924 (PMC13029173; doi:10.3390/nu18060924)
Supplement: Supplementary file 1 [file nutrients-18-00924-s001.zip › nutrients-4190043-supplementary.pdf]

Supplementary Material

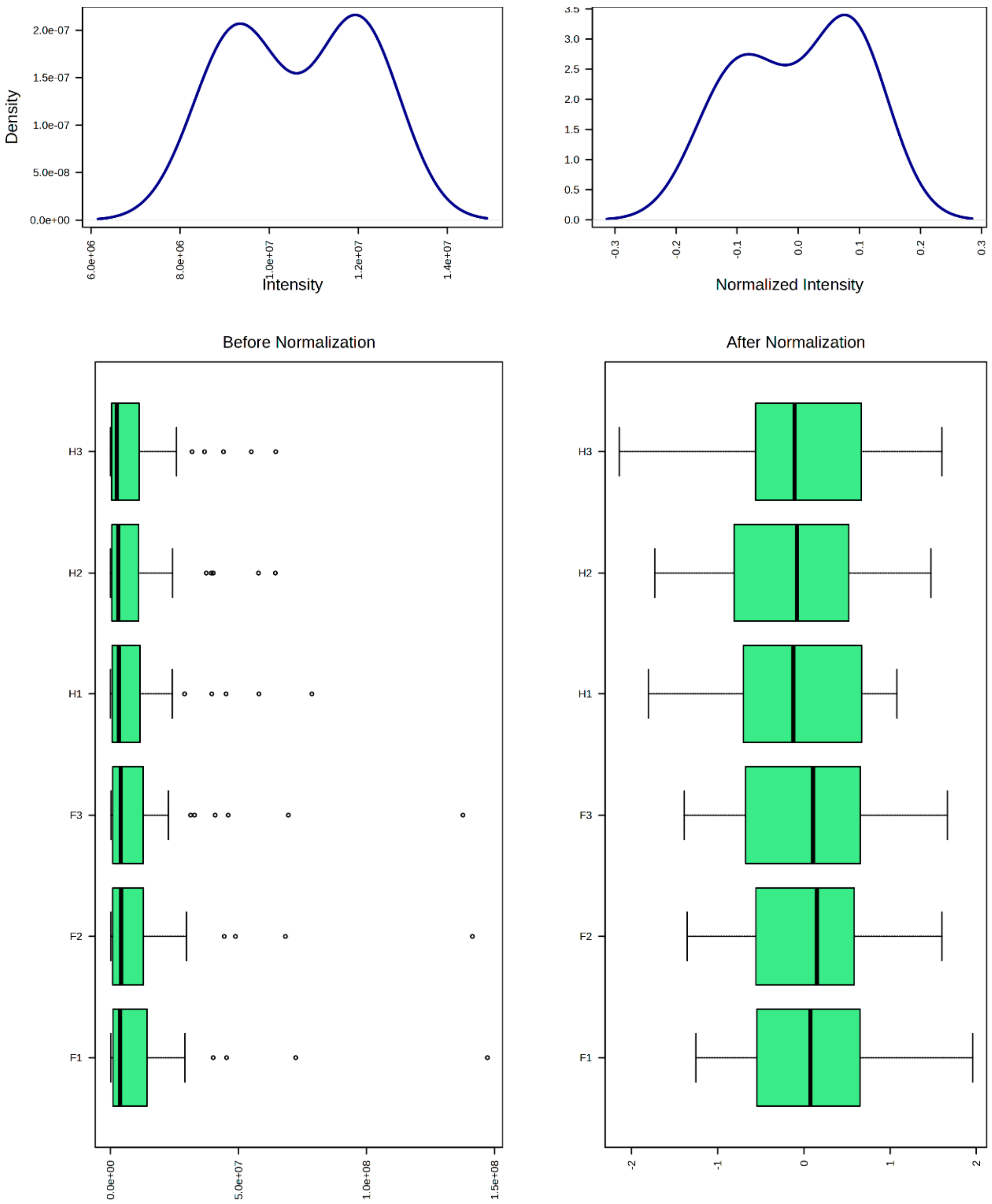

Figure S1. Distribution of metabolomic features before and after PQN normalization, log10 transformation, and Pareto scaling, visualized by boxplots and kernel density plots across all samples.
